# Stress and Anxiety to Viral Epidemics-6 for Medical Students: Psychometric Properties of the Anxiety Measure for the COVID-19 Pandemic

**DOI:** 10.3389/fpsyt.2021.705805

**Published:** 2021-08-03

**Authors:** Junseok Ahn, Jukab Lee, Youjin Hong, Jangho Park, Seockhoon Chung

**Affiliations:** ^1^Department of Psychiatry, Ulsan University Hospital, University of Ulsan College of Medicine, Ulsan, South Korea; ^2^Department of Psychiatry, GangNeung Asan Hospital, University of Ulsan College of Medicine, Gangneung, South Korea; ^3^Department of Psychiatry, Asan Medical Center, University of Ulsan College of Medicine, Seoul, South Korea

**Keywords:** stress, anxiety, psychological, health personnel, medical student, COVID-19

## Abstract

The aim of this study was to explore the psychometric properties and validity of Stress and Anxiety to Viral Epidemics-6 items (SAVE-6) among medical students who are at high risk of coronavirus disease 2019 (COVID-19) infection. A total of 212 medical students participated in the online anonymous survey that used SAVE-6, Coronavirus Anxiety Scale (CAS), Generalized Anxiety Disorder-7 items (GAD-7), and Work and Social Adjustment Scale (WSAS). We observed that the single-factor structure model of the SAVE-6 scale showed good internal consistency (Cronbach's alpha = 0.756) and a good convergent validity with GAD-7 (rho = 0.320, *p* < 0.001), CAS (rho = 0.229, *p* < 0.001), and WSAS (rho = 0.278, *p* < 0.001). The appropriate cut-off score of the SAVE-6 scale was determined as 15 points in accordance with at least a mild degree of generalized anxiety (GAD-7 score of 5) among medical students. In conclusion, the SAVE-6 scale can be applied to medical students as a reliable and valid rating scale to assess anxiety response to the present viral pandemic.

## Introduction

The ongoing global coronavirus disease 2019 (COVID-19) pandemic, which began in January 2020, has seized the entire world. In Korea, since the first confirmed case on January 20, 2020, 149,191 confirmed cases and 1,993 deaths have occurred as of June 2021^1^. Many patients have died in a psychiatric hospital in the neighboring area of Daegu city, which recorded a major breakout. Thereupon, patients as well as doctors, nurses, guardians, and other healthcare workers in the hospital were often infected by the virus. Since then, several hospitals conducted cohort quarantine or closed the emergency room that was occupied by the infected people for a certain period (1). Currently, a system of examination for classification and confirmation of patients has been moderately established; however, in the earlier days of the pandemic, healthcare workers experienced unprecedented quarantining (2). Despite the ongoing vaccination drives for healthcare workers as per the government's vaccination policy, they are still unable to be completely liberated from the anxiety of accidental exposure to the infection.

Like medical personnel, medical school students are also prone to anxiety regarding COVID-19 in hospitals since the onset of the COVID-19 pandemic (3). Although virtual classes were introduced as a part of social distancing, due to the nature of medical education, medical students were scheduled to meet for on-site education in venues like laboratory classes in basic medicine, clinical clerkship in training hospitals, and medical licensing examinations (4). Medical students are on the cusp of becoming medical experts as they are not yet certified medical professionals but are still trainees in the field (5). They may feel the responsibility of being medical experts, in spite of inadequate medical practice. Simultaneously, they constantly worry about spreading infection to their families or partners, similar to the general public. As can be seen in the medical students' syndrome, those who lack practical experience in the field are more vulnerable to worrying about diseases due to their inadequate knowledge of diseases or symptoms (6). As COVID-19 continues to progress, medical students are forced to overcome the fear of an uncertain disease and simultaneously face the difficulties of working in hospitals just like other medical personnel.

We developed the Stress and Anxiety to Viral Epidemics-9 items (SAVE-9) scale for measuring work-related stress and anxiety of healthcare workers in response to the ongoing viral pandemic (7). Since medical students are not healthcare workers, it is not appropriate to apply the SAVE-9 scale to them. We observed that the SAVE-9 scale was clustered into two factors: factor I—“anxiety about the viral epidemic” and factor II—“work-related stress associated with the viral epidemic.” We previously explored the validity of factor I (namely, SAVE-6) for measuring anxiety of the general population in Korea (8) and Lebanon (9). Several rating scales such as Coronavirus Anxiety Scale (CAS) (10), COVID-19-Anxiety Questionnaire (11), Fear of COVID-19 Scale (12), Obsession with COVID-19 Scale (13), Coronavirus Pandemic Anxiety Scale (14), COVID-19 Anxiety Syndrome Scale (15), and COVID-19 Anxiety Scale (16) were also developed and applied to assess the anxiety response of the general population to the viral epidemic. SAVE-6 included items inquiring about the apprehension of an individual during the current pandemic situation, worry about avoidance behavior of others, and concern about their own health and that of their family members (8, 9). In this study, we hypothesized that the SAVE-6 scale can be applied usefully to measure the anxiety response of medical students to the viral epidemic. Thus, we aimed to explore the psychometric properties and applicability of SAVE-6 among medical students who are at high risk of contracting COVID-19.

## Materials and Methods

### Participants and Procedure

This study was conducted through an online anonymous survey using Google Forms among medical students at the University of Ulsan College of Medicine (UUCM) between July 13 and August 1, 2020. The study protocol was approved and written informed consent for participation was waived by the Institutional Review Board (2020-1067) of the Asan Medical Center.

### Symptom Assessment

#### Stress and Anxiety to Viral Epidemics-6 Items

The Stress and Anxiety to Viral Epidemics-6 items (SAVE-6) scale is a version extracted from the original SAVE-9 scale (7) which was developed to assess work-related stress and anxiety of healthcare workers in response to the viral epidemic. The utility of SAVE-6 among the general population has been studied in Korea (8) and Lebanon (9). The items are rated on a 5-point Likert scale: 0 (never), 1 (rarely), 2 (sometimes), 3 (often), and 4 (always)^2^. A higher total score indicates a higher level of anxiety in response to the viral epidemic.

#### Generalized Anxiety Disorder 7 Items

The Generalized Anxiety Disorder-7 items (GAD-7) scale, a self-rating questionnaire, assesses general anxiety of people. It comprises seven items and is rated using a scale ranging from 0–3 (where, 0 = not at all, 3 = nearly every day), and the total score ranges from 0 to 21. A higher score reflects a more severe degree of anxiety symptoms (17). We used the Korean version of the GAD-7 scale^3^. In this sample, Cronbach's alpha = 0.894, and McDonald's omega = 0.903.

#### Coronavirus Anxiety Scale

The CAS scale screens anxiety and fear associated with COVID-19 in people (10). It consists of five items including dizziness, sleep disturbance, tonic immobility, appetite loss, and abdominal distress and is rated on a scale of 0–4 (0 = not at all, 4 = nearly every day). The Korean version of CAS was validated and used in this study (18). In this sample, Cronbach's alpha = 0.854, and McDonald's omega = 0.870.

#### Work and Social Adjustment Scale

The Work and Social Adjustment Scale (WSAS) examines functional impairment due to an identified psychiatric problem. It consists of five domains: (1) the ability to work or study, (2) home management, (3) social leisure activities, (4) private leisure activities, and (5) the ability to maintain close relationships. The WSAS is rated on a scale of 0–8 (0 = not at all, 8 = severely impaired) (19). In this study, we applied the Korean version of the WSAS that was created and translated with the author's permission in previous studies (18). In this sample, Cronbach's alpha = 0.717, and McDonald's omega = 0.776.

### Statistical Analysis

The SAVE-6 total score differences in gender (men vs. women), generalized anxiety (GAD-7 ≥ 5 vs. GAD-7 < 5), and functional impairment of mental health (WSAS ≥ 11 vs. WSAS < 11) were examined using independent *t*-tests. Correlations of the SAVE-6 total score with GAD-7, CAS, and WSAS were examined using Spearman's correlations, since the distributions of those scales scores were not within the normal limit. Before performing confirmatory factor analysis (CFA), the normality assumption of each of the six items was checked based on skewness and kurtosis for an acceptable limit of range ± 2 (20). The sampling adequacy and data suitability were examined using the Kaiser–Meyer–Olkin (KMO) test and Bartlett's test of sphericity. A bootstrap (2,000 samples) maximum likelihood CFA was conducted for the six items of SAVE-6 to explore the factorial validity for a unidimensional structure. Multi-group CFAs were run to examine whether SAVE-6 measures anxiety response the same way across gender (men vs. women), generalized anxiety (GAD-7 ≥ 5 vs. GAD-7 < 5), and functional impairment of mental health (WSAS ≥ 11 vs. WSAS < 11). Satisfactory model fit was defined by a standardized root-mean-square residual (SRMR) value ≤ 0.05, root-mean-square-error of approximation (RMSEA) value ≤ 0.10, comparative fit index (CFI) and Tucker Lewis index (TLI) values ≥ 0.90 (21, 22). The reliability and internal consistency of the factor was examined using Cronbach's alpha and McDonald's omega coefficients. Receiver operating characteristic (ROC) analysis was performed to explore the appropriate cut-off score of the SAVE-6 scale in accordance with generalized anxiety symptoms. Furthermore, we conducted an independent *t*-test and a chi-square test to examine the differences in clinical variables or rating scale scores using the IBM SPSS Statistics for Windows, Version 21.0 and AMOS version 27.

## Results

A total of 212 medical students in the UUCM participated in this survey (Table 1). Among them, 150 (70.8%) were men, and the proportions of students in each grade were similar. No students were infected, two of them had quarantine experience, and 15 (7.1%) of them reported having a past history of psychiatric symptoms.

**Table 1:** Demographic characteristics of participants (*N* = 212).

**Variables**	**Mean ± SD, *N* (%)**
**Gender (male)**	150 (70.8%)
**Grade**	
Pre-medicine 1st (UUCM, total *N* = 39)	36 (17.0%)
Pre-medicine 2nd (UUCM, total *N* = 46)	38 (17.9%)
Medicine 1st (UUCM, total *N* = 39)	30 (14.2%)
Medicine 2nd (UUCM, total *N* = 40)	32 (15.1%)
Medicine 3rd (UUCM, total *N* = 43)	44 (20.8%)
Medicine 4th (UUCM, total *N* = 40)	32 (15.1%)
**COVID-19 questions**	143 (15.3%)
Did you experience being quarantined due to infection with COVID-19? (Yes)	2 (0.9%)
Did you experience being infected with COVID-19? (Yes)	0 (0.0%)
**Psychiatric history**	
Have you experienced or been treated for depression, anxiety, or insomnia? (Yes)	15 (7.1%)
**Rating scales scores**	
Stress and anxiety to viral epidemics-6 items (SAVE-6)	11.0 ± 4.5 (0–23)
Generalized anxiety disorder-7 items (GAD-7)	1.9 ± 3.0 (0–17)
Coronavirus anxiety scale (CAS)	0.3 ± 1.2 (0–10)
Work and social adjustment scale (WSAS)	8.5 ± 6.2 (0–28)

*SD, standard deviation; UUCM, University of Ulsan College of Medicine*.

### Factor Structure of the Stress and Anxiety to Viral Epidemics-6 Items Among Medical Students

The normality assumption for the six items of SAVE-6 were checked using the skewness and kurtosis values, and we accepted values ranged within ± 2 (Table 2). Before the factor analysis, we checked sampling adequacy and data suitability, and observed that the KMO measure was 0.79 and Bartlett's test of sphericity was *p* < 0.001. CFA showed a single-factor model with good fit for all indices (CFI = 0.96; TLI = 0.94; SRMR = 0.04; RMSEA = 0.07), and these results supported the factorial validity of the SAVE-6 scale. Multi-group CFAs were conducted to test whether SAVE-6 measured the same way across gender (men vs. women), anxiety (GAD-7 ≥ 5 vs. GAD-7 < 5), and functional impairment of mental health (WSAS ≥ 11 vs. WSAS < 11). The results showed no differences in gender (CFI = 0.96; TLI = 0.94; SRMR = 0.04; RMSEA = 0.07), anxiety (CFI = 0.96; TLI = 0.93; SRMR = 0.04; RMSEA = 0.07), and mental health (CFI = 0.94; TLI = 0.91; SRMR = 0.06; RMSEA = 0.09), which demonstrated that measurement invariance was not observed when we measured anxiety response across gender, anxiety, or mental health using the SAVE-6 scale.

**Table 2:** Factor structure of the stress and anxiety to viral epidemics-6 items applied to medical students.

**Items**	**Responses**					**Mean ± SD**	**Skewness**	**Kurtosis**	**CID**	**Factor loading**
	**0**	**1**	**2**	**3**	**4**					
1. Are you afraid the virus outbreak will continue indefinitely?	2.4%	9.0%	17.9%	53.8%	17.0%	2.74 ± 0.93	−0.871	0.645	0.759	0.446
2. Are you afraid your health will worsen because of the virus?	21.7%	34.4%	26.9%	13.7%	3.3%	1.42 ± 1.08	0.405	−0.546	0.756	0.610
3. Are you worried that you might get infected?	19.8%	35.4%	19.3%	20.8%	4.7%	1.55 ± 1.16	0.349	−0.881	0.694	0.736
4. Are you more sensitive toward minor physical symptoms than usual?	17.5%	19.8%	17.5%	35.4%	9.9%	2.00 ± 1.29	−0.224	−1.169	0.677	0.804
5. Are you worried that others might avoid you even after the infection risk has been minimized?	45.8%	36.8%	5.2%	9.9%	2.4%	0.86 ± 1.05	1.288	0.899	0.744	0.569
6. Do you worry your family or friends may become infected because of you?	11.3%	9.9%	19.3%	44.8%	14.6%	2.42 ± 1.19	−0.729	−0.367	0.707	0.697

*Cronbach's alpha = 0.756*.

*SD, standard deviation; SAVE-6, Stress and Anxiety to Viral Epidemics-6 items; CID, Cronbach's alpha if item is deleted*.

*0 = never; 1 = rarely; 2 = sometimes; 3 = often; 4 = always*.

### Reliability and Evidence-Based Relationship With Other Variables

The SAVE-6 scale showed good internal consistency among medical students (Cronbach's alpha = 0.756, and McDonald's omega = 0.773), and it was similar to the Cronbach's alphas if each item was deleted (0.677–0.759, Table 2). SAVE-6 had a good convergent validity based on Spearman correlation coefficient with GAD-7 (rho = 0.320, *p* < 0.001), CAS (rho = 0.229, *p* < 0.001), and WSAS (rho = 0.278, *p* < 0.001). The SAVE-6 scale score was significantly higher among female students [*t*_(210)_ = 3.573, *p* < 0.001], with generalized anxiety {GAD-7 ≥ 5, [*t*_(210)_ = 3.396, *p* < 0.001]} and functional impairment of mental health {WSAS ≥ 11, [*t*_(210)_ = 3.387, *p* < 0.001]}.

### Cut-Off Score for Stress and Anxiety to Viral Epidemics-6 Items Among Medical Students

ROC analysis was conducted to explore the appropriate cut-off score of the SAVE-6 scale among medical students in accordance with at least a mild degree of generalized anxiety (GAD-7 score of 5). We observed that a score of 15 points was appropriate (area under the curve = 0.657, sensitivity = 0.51, specificity = 0.77).

## Discussion

In this study, we explored the psychometric properties and convergent validity of SAVE-6 among medical students and observed that the single-factor structure of SAVE-6 showed good internal consistency and convergent validity with other anxiety scales like GAD-7 and CAS. Furthermore, the appropriate cut-off score of the SAVE-6 scale was determined as 15 with at least a mild degree of generalized anxiety (GAD-7 score of 5) among medical students.

The SAVE-9 scale was originally developed to assess healthcare workers' stress and anxiety to the viral epidemic. Before commencing the study, we considered applying the SAVE-9 scale to medical students, since they stayed at the hospital and occasionally performed patient care roles. However, we decided to use SAVE-6, which was originally meant to be applied to the general population, since SAVE-9 had a few items that were not applicable to medical students. First, item 9 of SAVE-9, “Do you think that your colleagues would have more work to do due to your absence from a possible quarantine and might blame you?” was not appropriate for medical students, since they do not work professionally and thus are not replaced by other medical students. Additionally, item 6, “Do you feel skeptical about your job after going through this experience?” is also not applicable to medical students. Clinical clerkship is not a “job” for medical students, as they will begin working professionally as doctors in the future. Furthermore, item 7, “After this experience, do you think you will avoid treating patients with viral illnesses?” can be a question about “selecting their majors after getting a medical license.”

In this study, a single-structure model of SAVE-6 was confirmed to be valid and in line with previous studies (8, 9). However, we observed a relatively low factor loading value of 0.446 for item 1, “Are you afraid that the virus outbreak will continue indefinitely?” The survey was conducted during the summer of 2020, when people were worried that the COVID-19 pandemic would be difficult to control, which may have contributed to the high proportion of answers of “often” (53.8%) and “always” (17.0%) among medical students. In this model, values >0.6 for factor loading are acceptable. However, a value of < 0.5 is also acceptable when the composite reliability is higher than 0.6 (23). In this study, we observed good reliability (Cronbach's alpha = 0.756, and McDonald's omega = 0.773), thus this model was accepted. Furthermore, the reliability was not significantly more even if the item 1 was deleted (Table 2). The factor loading value was < 0.6 for item 5, “Are you worried that others might avoid you even after the infection risk has been minimized?”, due to the high proportion of the responses of “never” (45.8%) and “rarely” (36.8%). This result was similar to that of our previous study of SAVE-6 applied to the general Korean population (8).

The appropriate cut-off score for the SAVE-6 scale in accordance with at least a mild degree of GAD-7 was determined to be 15 among the participants of this study. In our previous studies, we observed the same results among the general population (8) and healthcare workers (factor I of the SAVE-9 scale) (7). Although it can vary depending on groups, races, or regions, SAVE-6 can be a useful tool for measuring the anxiety response of medical students to a viral epidemic like the current COVID-19 pandemic.

The anonymous online survey method is a limitation of this study. Amid the ongoing pandemic, we conducted an online survey to prevent the spread of the viral infection. Among the participants, no one was infected, and only two experienced quarantine. This low proportion of actual infection experience or quarantine may have influenced the results. Insufficient sample size is also one of the limitations of this study. Another limitation is that rating scales in this study were not formally validated for medical students. Additionally, the participation of UUHM students and the uneven gender ratio of the sample can be regarded as limitations. Furthermore, information about the participants' ages was not recorded since all participants were in their 20s, and we grouped them based on their grades and not age. In conclusion, the SAVE-6 scale can be applied to medical students as a reliable and valid rating scale to assess anxiety response to the COVID-19 pandemic.

## Data Availability Statement

The raw data supporting the conclusions of this article will be made available by the authors, without undue reservation.

## Ethics Statement

The studies involving human participants were reviewed and approved by Institutional Review Board (2020-1067) of the Asan Medical Center. The patients/participants provided their written informed consent to participate in this study.

## Author Contributions

JA drafted or provided critical revision of the article. JA and JL performed experiments and analysis. JL provided final approval of the version to publish. YH agreed to be accountable for all aspects of the work in ensuring that questions related to the accuracy or integrity of any part of the work are appropriately investigated and resolved. SC and JP contributed substantially to the conception and design of the study, the acquisition of data, and or the analysis and interpretation. All authors contributed to the article and approved the submitted version.

## Conflict of Interest

The authors declare that the research was conducted in the absence of any commercial or financial relationships that could be construed as a potential conflict of interest.

## Publisher's Note

All claims expressed in this article are solely those of the authors and do not necessarily represent those of their affiliated organizations, or those of the publisher, the editors and the reviewers. Any product that may be evaluated in this article, or claim that may be made by its manufacturer, is not guaranteed or endorsed by the publisher.

## Footnotes

^1^Available online at: http://ncov.mohw.go.kr/en/ (accessed June 17, 2021).

^2^Available online at: https://www.save-viralepidemic.net (accessed June 17, 2021).

^3^Available online at: https://www.phqscreeners.com/ (accessed June 17, 2021).

## Abbreviations

CAS: Coronavirus Anxiety Scale
CFA: confirmatory factor analysis
CFI: comparative fit index
CID: Cronbach's alpha if item is deleted
GAD-7: Generalized Anxiety Disorder-7 items
KMO: Kaiser–Meyer–Olkin
RMSEA: root-mean-square-error of approximation
ROC: receiver operating characteristic
SAVE-6: Stress and Anxiety to Viral Epidemics-6 items
SRMR: standardized root-mean-square residual
TLI: Tucker Lewis index
UUCM: University of Ulsan College of Medicine
WSAS: Work and Social Adjustment Scale.
